# Modulation of complement activation by pentraxin-3 in prostate cancer

**DOI:** 10.1038/s41598-020-75376-z

**Published:** 2020-10-27

**Authors:** Giovanni Stallone, Giuseppe Stefano Netti, Luigi Cormio, Giuseppe Castellano, Barbara Infante, Paola Pontrelli, Chiara Divella, Oscar Selvaggio, Federica Spadaccino, Elena Ranieri, Francesca Sanguedolce, Antonio Pennella, Loreto Gesualdo, Giuseppe Carrieri, Giuseppe Grandaliano

**Affiliations:** 1 Nephrology Dialysis and Transplantation Unit, University of Foggia, Viale Luigi Pinto, 251, 71122 Foggia, Italy; 2 Clinical Pathology Unit, Department of Medical and Surgical Sciences, University of Foggia, Viale Luigi Pinto, 251, 71122 Foggia, Italy; 3 Urology and Renal Transplantation Unit, University of Foggia, Viale Luigi Pinto, 251, 71122 Foggia, Italy; 4 Nephrology, Dialysis and Transplantation Unit, Department of Emergency and Organ Transplantation, University of Bari “Aldo Moro”, Bari, Italy; 5 Pathology Unit, Department of Clinical and Experimental Medicine, University of Foggia, Viale Luigi Pinto, 251, 71122 Foggia, Italy; 6 Fondazione Policlinico Universitario A. Gemelli IRCCS, Largo A. Gemelli 8, 00168 Rome, Italy; 7 Università Cattolica del Sacro Cuore, Largo F. Vito 1, 00168 Rome, Italy; 8 Nephrology, Dialysis and Transplantation Unit, Department of Medical and Surgical Sciences, University of Foggia, Viale Luigi Pinto, 251, 71122 Foggia, Italy

**Keywords:** Cancer, Immunology

## Abstract

Pentraxin 3 (PTX3) is an essential component of the innate immune system and a recognized modulator of Complement cascade. The role of Complement system in the pathogenesis of prostate cancer has been largely underestimated. The aim of our study was to investigate the role of PTX3 as possible modulator of Complement activation in the development of this neoplasia. We performed a single center cohort study; from January 2017 through December 2018, serum and prostate tissue samples were obtained from 620 patients undergoing prostate biopsy. A group of patients with benign prostatic hyperplasia (BPH) underwent a second biopsy within 12–36 months demonstrating the presence of a prostate cancer (Group A, n = 40) or confirming the diagnosis of BPH (Group B, N = 40). We measured tissue PTX3 protein expression together with complement activation by confocal microscopy in the first and second biopsy in group A and B patients. We confirmed that that PTX3 tissue expression in the first biopsy was increased in group A compared to group B patients. C1q deposits were extensively present in group A patients co-localizing and significantly correlating with PTX3 deposits; on the contrary, C1q/PTX3 deposits were negative in group B. Moreover, we found a significantly increased expression of C3a and C5a receptors within resident cells in group A patient. Interestingly, C1q/PTX3 deposits were not associated with activation of the terminal Complement complex C5b-9; moreover, we found a significant increase of Complement inhibitor CD59 in cancer tissue. Our data indicate that PTX3 might play a significant pathogenic role in the development of this neoplasia through recruitment of the early components of Complement cascade with hampered activation of terminal Complement pathway associated with the upregulation of CD59. This alteration might lead to the PTX3-mediated promotion of cellular proliferation, angiogenesis and insensitivity to apoptosis possible leading to cancer cell invasion and migration.

## Background

Prostate cancer is the second leading cause of cancer-related death in males^1^. The hormonal milieu has always been considered to play a key role in the pathogenesis of this neoplasia^2^, although an increasing interest has been focused in the last decade on the role of chronic inflammation in this setting. Indeed, a growing body of evidence suggests that chronic prostate chronic inflammation is significantly associated with a higher incidence rate of prostate cancer^2^, but the fine molecular mechanisms of this link are still largely unclear. Pentraxin-3 (PTX3), as most of the proteins belonging to the pentraxin superfamily, is produced in response to inflammatory triggers^3^. Interestingly, PTX3 has been shown to facilitate the dysregulation of mitogenic signaling pathways and to promote cellular proliferation, angiogenesis, insensitivity to apoptosis, cancer cell invasion and migration, and tumor escape from immunosurveillance^4–7^. We have previously demonstrated that an increased PTX3 prostate expression and PTX3 serum levels may predict the subsequent development of prostate cancer^7^.

It is well known that PTX3 may play a pivotal role in the regulation of Complement cascade activation^3,5,6^ and the literature provides several evidences of an insidious relationship between Complement activation and cancer in terms of cellular proliferation and angiogenesis^8–11^.

In this scenario, a particular interest has been showed by Complement proteins C3a and C5a. Indeed, several reports documented the proliferative abilities of both anaphylatoxins^6,12,13^ through the activation of different signal transduction pathways linked with cancer progression. C3a (C3aR) and C5a receptor (C5aR) are G-proteins-coupled receptors^14,15^ activating different members of the mitogen-activated protein kinase family including extracellular signal-regulated kinases and p38^16–18^. Furthermore, C3a and C5a increase the activation of phosphatidylinositol 3-kinase, Akt, and mammalian target of rapamycin^19,20^, three signal transduction proteins strongly associated with neoplasia^21,22^. O'Barr et al. demonstrated that C3a/C3aR promotes proliferation of undifferentiated human neuroblastoma cells through protein kinase C and NF-κB activation^23^. Noteworthy, complement proteins can directly and indirectly play a role in the pathogenesis of neoplastic angiogenesis. Indeed, both anaphylatoxins along with C1q have been shown to directly influence endothelial cells proliferation and migration^24^. On the other hand, the same Complement cascade components can elicit a pro-angiogenic phenotype in infiltrating tissue macrophages^25^.

Considering the limited data on the role of Complement activation in the development of prostate cancer, the aims of present study were to elucidate the potential relationship between PTX3 and Complement cascade activation in this contest. In particular, we studied whether increased PTX3 expression was associated with Complement cascade activation and whether the latter event may be related with progression from chronic prostate inflammation to prostate cancer.

## Materials and methods

### Study population

The population of the present observational, single center, cohort study consisted of 620 consecutive patients who received a first prostate biopsy (PBx) from January 1st 2017 to December 31th 2018 at the Urology Unit of the University Hospital “Policlinic Riuniti” of Foggia, Italy. The patients included in the study received an 18-core biopsy scheme following local anesthesia, as previously described^26^. Histological diagnosis included benign prostatic hyperplasia (BPH) (n = 385) and prostate cancer (n = 235). A serum sample was obtained from each patient at the time of biopsy and stored at − 80 °C. One hundred and twenty-seven patients with a histological diagnosis of BPH received within 12–36 months a second PBx as clinically indicated. Forty of them were diagnosed with prostate cancer at the second biopsy (group A). A matched group of patients, with a second negative biopsy performed at least one year after the first negative one, (group B, n = 40) was identified using a propensity score multidimensional matching technique based on the following clinical variables: age, serum PSA value, prostate volume, Digital Rectal Exploration findings, and time to second biopsy.

Written informed consent to take part was given by all participants. The protocol for the research project has been approved by the local Ethics Committee (Decision n. 152/CE/2014 of September 03, 2014; Ethical Committee at the University Hospital “Ospedali Riuniti” of Foggia) and conforms to the guidelines laid down by the Regional Ethics Committee on human experimentation and to the provisions of the Declaration of Helsinki in 1995.

### Histologic analysis of prostate tissue

Two senior uropathologists performed the analysis using contemporary diagnostic criteria for prostatic intraepithelial neoplasia (PIN), atypical small acinar proliferation (ASAP) and prostatic cancer (PCa). Moreover, biopsy samples of both benign prostatic tissue and PCa were graded on a 4-point scale for inflammation (0-no inflammatory cells, 1-scattered inflammatory cell infiltrate, 2-non-confluent lymphoid nodules and 3-large inflammatory areas with confluence of infiltrate) and aggressiveness (0-no contact between inflammatory cells and glandular epithelium; 1-contact between inflammatory cell infiltrate and glandular epithelium; 2-clear but limited, less than 25% of the examined material, glandular epithelium disruption, and 3-glandular epithelium disruption more than 25% of the examined material), as described by Irani et al.^27^.

### Indirect immunofluorescence and confocal laser scanning microscopy

A double-label immunofluorescence was performed to evaluate the expression of PTX-3, C1q, MBL, C3aR, C5aR1, C5b-9 and CD59 and their eventual co-localization. To this purpose we employed the following primary antibodies: rat monoclonal IgG2a anti-PTX-3 antibody (clone MNB4, Abcam, Cambridge UK)^28,29^, mouse monoclonal IgG1/k anti-PSA antibody (clone ER-PR8, Dako-Agilent, Santa Clara, CA), mouse monoclonal IgG2b anti-C1q (clone JL-1; Abcam); rabbit monoclonal IgG anti-Mannose Binding Lectin (anti-MBL) (clone EPSISR5; Abcam); rabbit polyclonal IgG anti-C3aR (Abcam); mouse monoclonal IgG2a anti-C5R1/CD88 (clone P12/1; Abcam); mouse monoclonal IgG2a anti-C5b-9 (clone aE11; Abcam); rabbit polyclonal IgG anti-CD59 (Sigma-Merck KGaA, Darmstadt, Germany).

After paraffin removal, tissue sections were incubated at 4 °C overnight with a mixture of primary antibodies diluted 1:100 in PBS pH 7.4. The immune complexes were detected using the Alexa-Fluor 488 goat anti-rat and 546 goat anti-mouse IgG and 546 goat anti-rabbit IgG (all from Alexa, Thermo Fisher, Waltham, MA). Negative controls were obtained incubating serial sections with the blocking solution and irrelevant antibody.

After washing in PBS (3 × 5′) the sections and the negative control were incubated 1 h at room temperature with goat anti-rat IgG 488 and goat anti-mouse IgG 546 or goat anti-rabbit IgG 546, as appropriate. All secondary antibodies were used at a dilution of 1:250.

To stain the nuclei, after washing in PBS pH 7.4 (3 × 5′) samples were incubated with TO-PRO diluted 1:5000 in PBS pH 7,4 (Invitrogen-Molecular Probe, Thermo Fisher, Waltham, MA). The slides were mounted in Gel Mount (Sigma) and sealed.

Specific fluorescence was evaluated by confocal microscopy using the Leica TCS SP5 (Leica, Wetzlar, Germany) equipped with argon-krypton (488 nm), green-neon (543 nm), and helium–neon (633 nm) lasers. Fluorescence quantification was performed as previously described^7^.

### Statistical analysis

Statistical analysis was performed using SPSS 25.0 software (SPSS Inc, Evanston, IL). Normality of variable distribution was tested using Kolmogorov–Smirnov test. Comparison of variables between the different groups was obtained with Student’s t test and Mann–Whitney U test owing to normal or non-parametric distribution. Sperman’s non parametric test was used to investigate the correlation between PTX3 and C1q deposits. A *p* value of ≤ 0.05 was considered statistically significant. Results are expressed in the text as mean ± standard deviation, unless otherwise stated.

## Results

**Figure 1:**
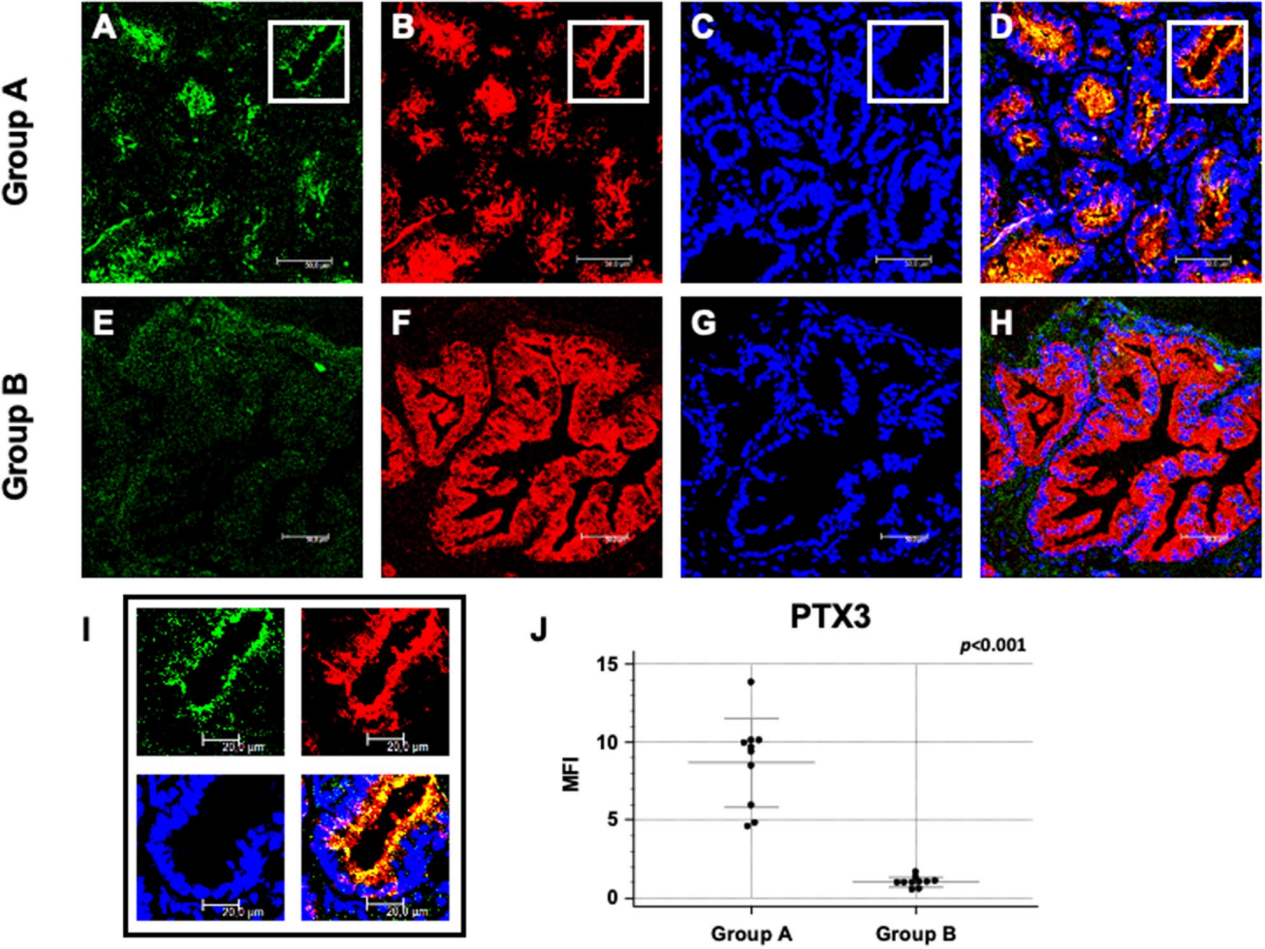
PTX3 protein expression and co-localization with PSA in first PBx of patients subsequently developing prostate cancer (group A) or not (group B). PTX3 (green; **A** and **E**) and PSA (red; **B** and **F**) protein expression were evaluated in the first PBx by double label immunofluorescence and confocal microscopy as detailed in Materials and Methods. TO-PRO-3 was used to stain nuclei (blue; **C** and **G**). Merged images (**D** and **H**) demonstrate the co-localization of the two protein signals (yellow). Mean fluorescence intensity (MFI) quantification confirmed a statistically significant higher PTX3 expression in group A compared to group B (**I**, **J**). Bar length = 50 µ.

We first evaluated PTX3 expression in the first biopsy of the two groups of patients undergoing a second PBx within 12–36 months from the first one, to confirm in this new larger cohort our previous findings. As already described^7^, PTX3 tissue expression was significantly higher in group A (Fig. 1A–D) then in group B patients (Fig. 1E–H). This observation was strengthened by quantification of specific fluorescence (Fig. 1I,J). We also confirmed that PTX3 protein expression extensively colocalized with PSA (Fig. 1A–H).

**Figure 2:**
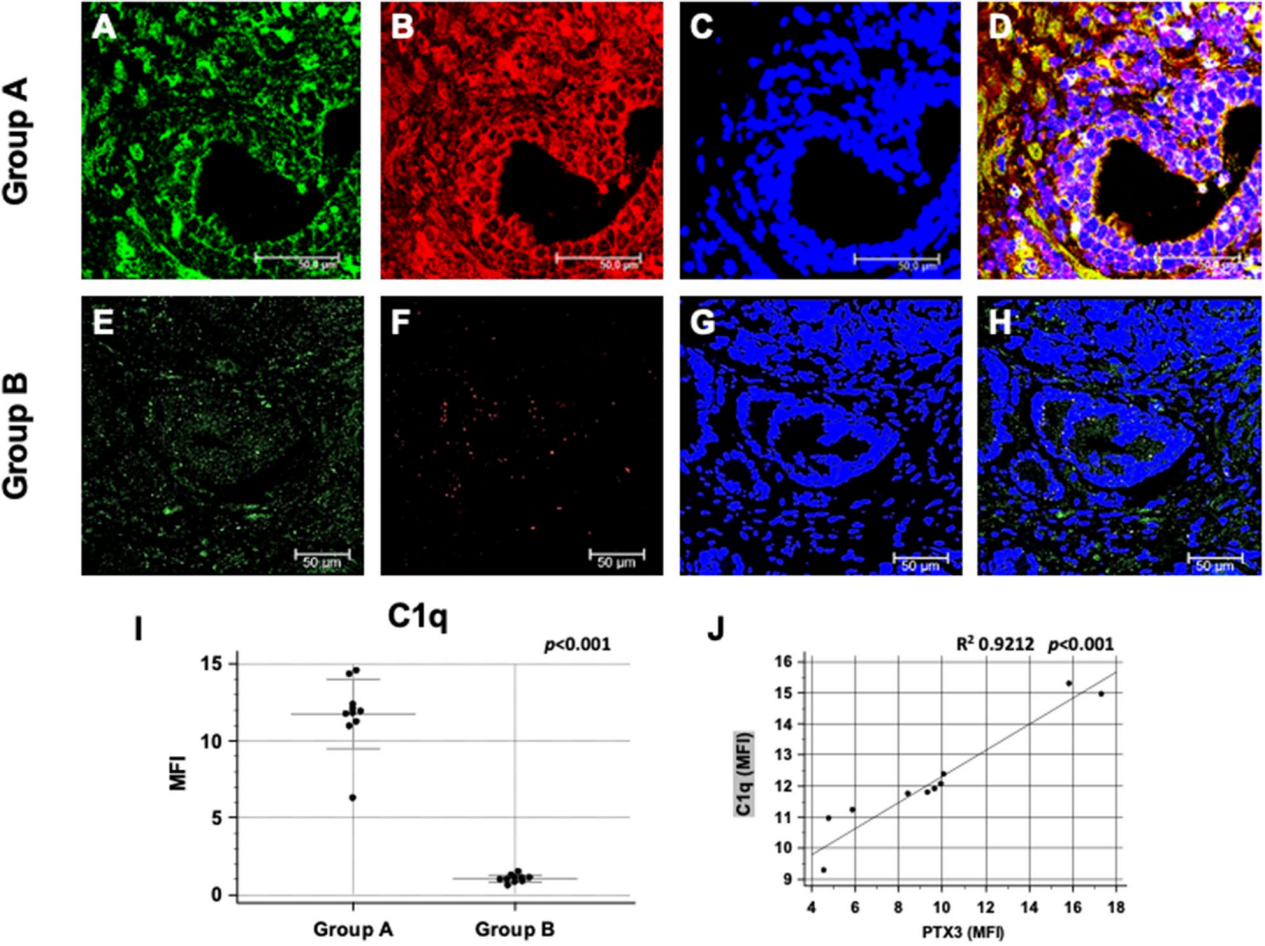
Complement factor C1q deposition and co-localization with PTX3 in first PBx of patients subsequently developing prostate cancer (group A) or not (group B). PTX3 protein expression (green; **A** and **E**) and C1q deposition (red; **B** and **F**) were evaluated in the first PBx by double label immunofluorescence and confocal microscopy as detailed in Materials and Methods. TO-PRO-3 was used to stain nuclei (blue; **C** and **G**). Merged images (**D** and **H**) demonstrate the co-localization of the two protein signals (yellow). Mean fluorescence intensity (MFI) quantification confirmed a statistically significant higher C1q deposition in group A compared to group B (**I**). Bar length = 50 µ; (**J**) significant correlation of PTX3 and C1q deposits in group A.

We then investigated the activation of the Complement cascade in the first biopsy of both study groups. Since PTX3 can activate the Complement system through the classic pathway, we evaluated the deposition of C1q. Interestingly, C1q deposition was extensively present in tissue samples from group A patient (Fig. 2A–D), whereas was completely absent in-group B (Fig. 2E–H), in accordance to PTX3 expression levels. On the other hand, the deposition of MBL, one of the recognition molecules in the lectin pathway of Complement cascade activation, was absent in both groups (data not shown). The quantification of C1q specific immunofluorescence clearly demonstrated a statistically significant difference between the two study groups (Fig. 2I,J). Interestingly, double immunofluorescence clearly demonstrated a co-localization C1q/PTX3 in-group A tissue samples (Fig. 2A–D). Interestingly, we found a correlation between PTX3 and C1q deposits in patients of group A who developed prostate cancer (Fig. 2J).

**Figure 3:**
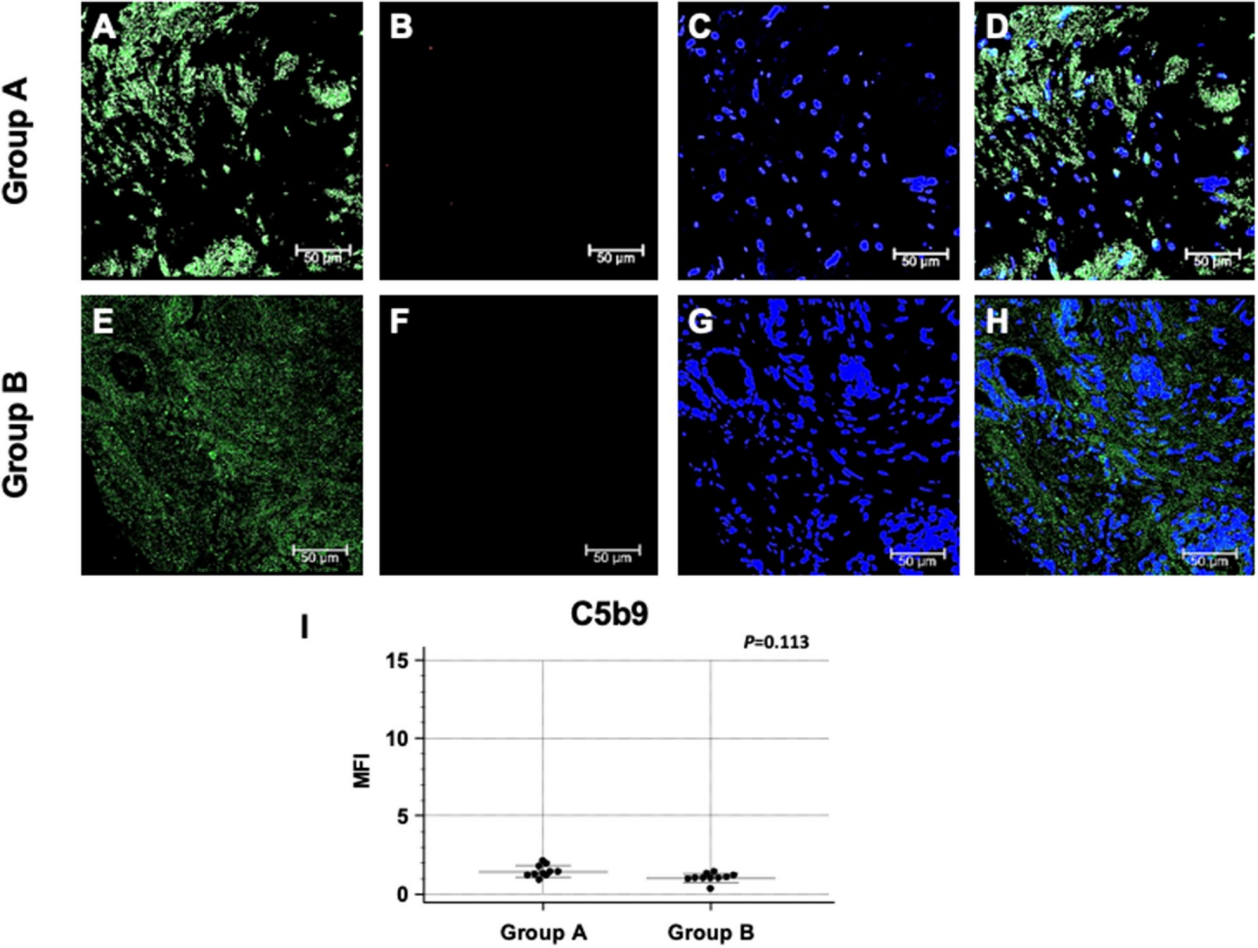
Terminal Complement complex, C5b-9, deposition and co-localization with PTX3 in first PBx of patients subsequently developing prostate cancer (group A) or not (group B). PTX3 protein expression (green; **A** and **E**) and C5b-9 deposition (red; **B** and **F**) were evaluated in the first PBx by double label immunofluorescence and confocal microscopy as detailed in Materials and Methods. TO-PRO-3 was used to stain nuclei (blue; **C** and **G**). Merged images (**D** and **H**) do not demonstrate any co-localization of the two protein signals. Mean fluorescence intensity (MFI) quantification confirmed the absence of C5b-9 deposition in both groups (**I**). Bar length = 50 µ.

To further investigate the Complement cascade, we next evaluated the tissue deposition of the terminal Complement complex, C5b-9. Surprisingly, the increased expression of C1q did not correspond to an increased deposition of the terminal Complement complex. Indeed, C5b-9 specific immunofluorescence was completely absent in the prostate tissue of the first biopsy of both group A and B patients (Fig. 3A–I).

**Figure 4:**
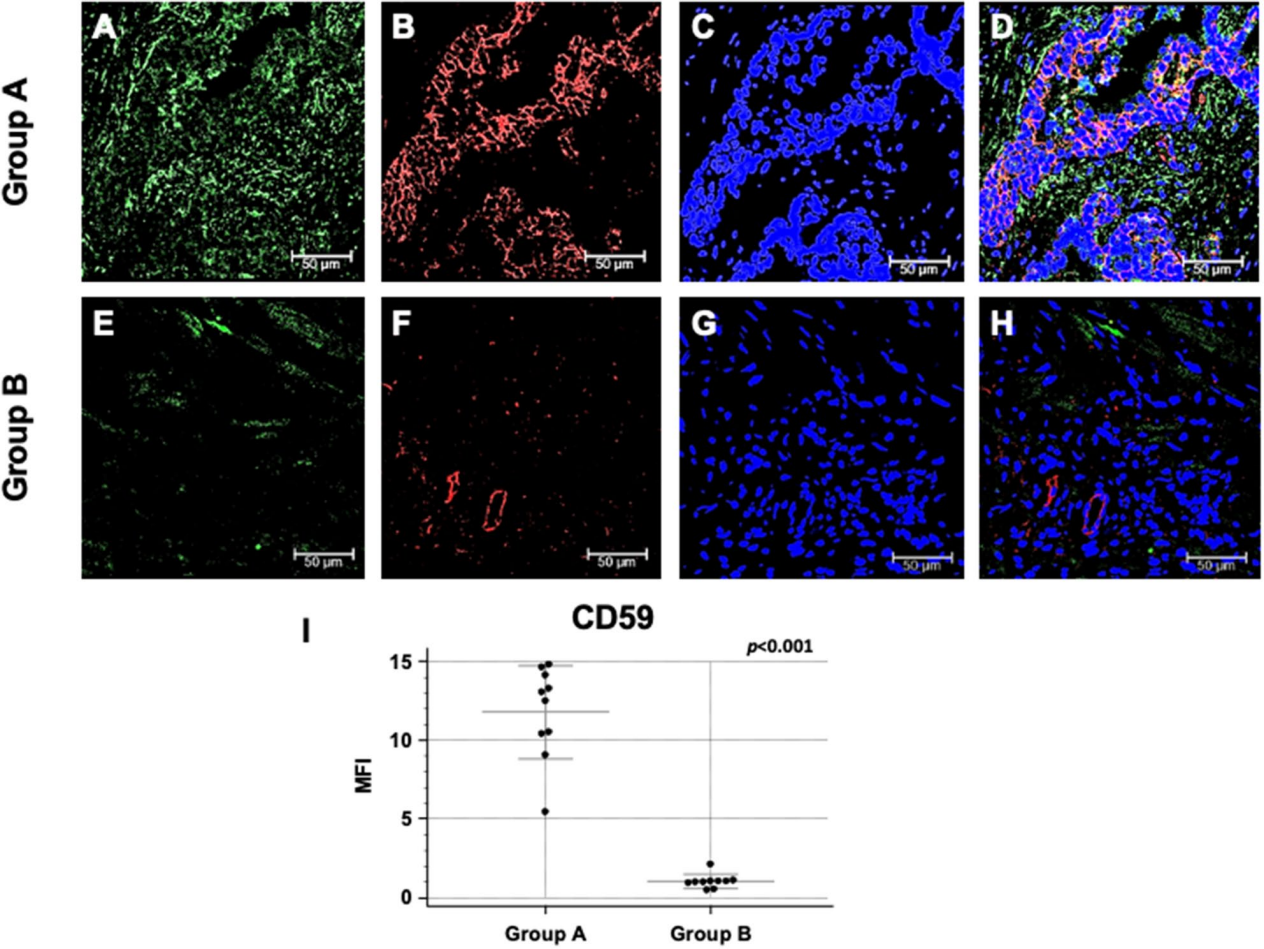
CD59 protein expression and co-localization with PTX3 in first PBx of patients subsequently developing prostate cancer (group A) or not (group B). Protein expression of PTX3 (green; **A** and **E**) and CD59 (red; **B** and **F**) were evaluated in the first PBx by double label immunofluorescence and confocal microscopy as detailed in Materials and Methods. TO-PRO-3 was used to stain nuclei (blue; **C** and **G**). Merged images (**D** and **H**) demonstrate the co-localization of the two protein signals (yellow). Mean fluorescence intensity (MFI) quantification confirmed a statistically significant higher CD59 expression in group A compared to group B (**I**). Bar length = 50 µ.

In order to investigate the possible inhibition of the activation of the Complement cascade, we evaluated the expression of CD59, one of the complement cascade inhibitors^13^ that can prevent C5b-9 assembly^10,30^. Interestingly, an increased CD59 expression has been reported in several neoplasia^31^, although no information is available on the level of CD59 expression within neoplastic or inflamed prostate tissue. Noteworthy, CD59 protein expression was markedly increased in PBx of group A compared with group B patients, mainly within resident cells (Fig. 4A–H). Quantification of CD59 specific immunofluorescence demonstrated that the difference between the two study groups were statistically significant (Fig. 4I).

**Figure 5:**
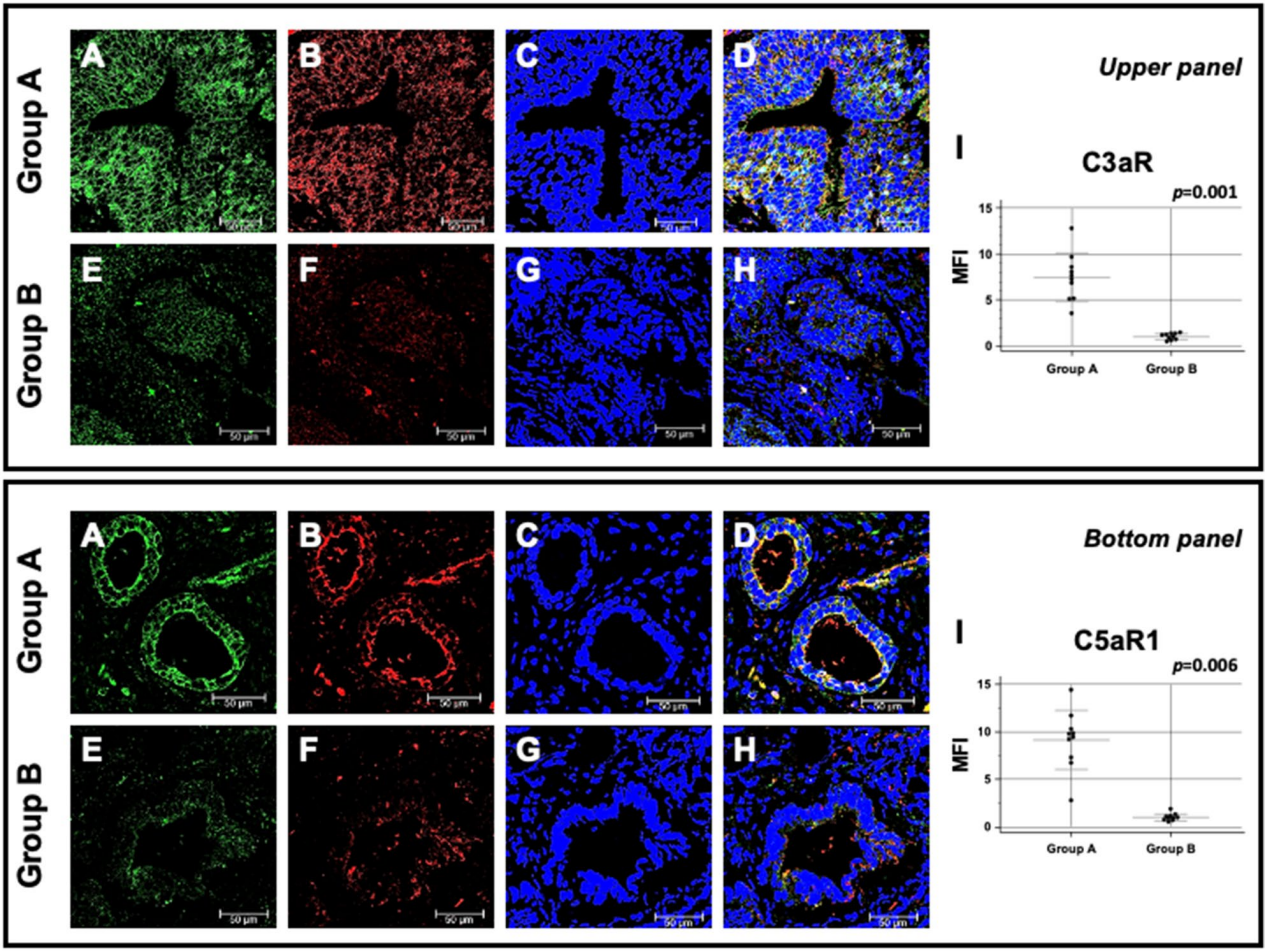
C3a and C5a receptors protein expression and co-localization with PTX3 in first PBx of patients subsequently developing prostate cancer (group A) or not (group B). UPPER PANEL: Protein expression of PTX3 (green; **A** and **E**) and C3a receptor (red; **B** and **F**) were evaluated in the first PBx by double label immunofluorescence and confocal microscopy as detailed in Materials and Methods. TO-PRO-3 was used to stain nuclei (blue; **C** and **G**). Merged images (**D** and **H**) demonstrate the co-localization of the two protein signals (yellow). Mean fluorescence intensity (MFI) quantification confirmed a statistically significant higher C3a receptor expression in group A compared to group B (I). Bar length = 50 µ. BOTTOM PANEL: Protein expression of PTX3 (green; **A** and **E**) and C5aR1 (red; **B** and **F**) were evaluated in the first PBx by double label immunofluorescence and confocal microscopy. TO-PRO-3 was used to stain nuclei (blue; **C** and **G**). Merged images (**D** and **H**) demonstrate the co-localization of the two protein signals (yellow). Mean fluorescence intensity (MFI) quantification confirmed a statistically significant higher C5a receptor expression in group A compared to group B (**I**). Bar length = 50 µ.

Since anaphylatoxins were suggested as possible soluble mediators modulating both cancer cell proliferation and neoplastic angiogenesis^11^, we investigated the protein expression of C3a and C5a receptors. The expression of both trans-membrane proteins was markedly up-regulated in the first PBx of group A compared to group B patients (Fig. 5 upper panel A–H and Fig. 5 bottom panel A–H). Also, in this case, the quantification of specific fluorescence, confirmed that the difference was statistically significant (Fig. 5I upper panel and Fig. 5I bottom panel). Remarkably, the expression of CD59, C3aR and C5aR1 co-localized within the prostate tissue samples with PTX3 expression (Figs. 4D, 5D upper panel and 5D bottom panel respectively).

**Figure 6:**
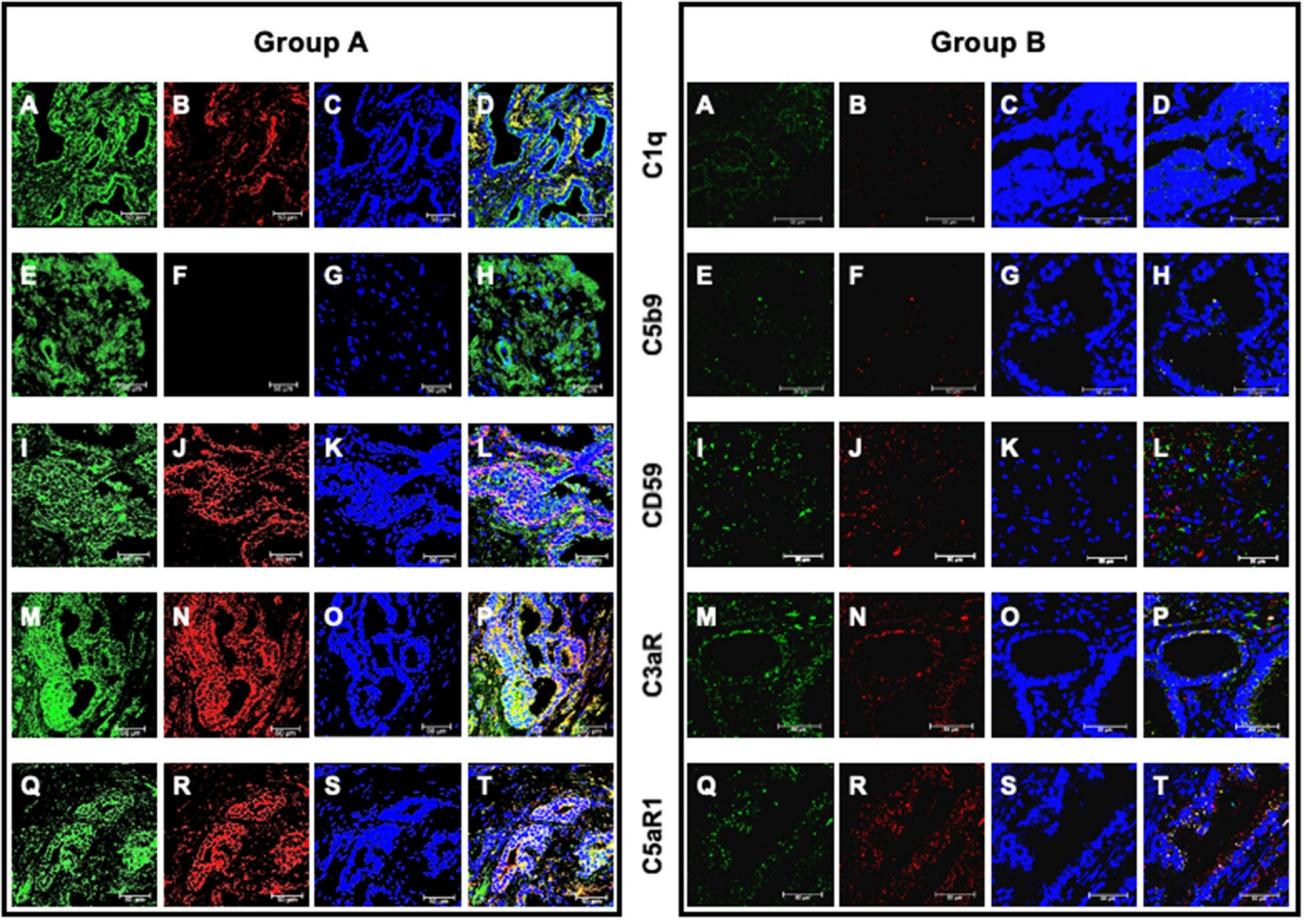
C1q and C5b-9 deposition and CD59, C3a and C5a receptor expression and their co-localization with PTX3 in second biopsy of patients who developed prostate cancer (group A) or not (group B). PTX3 (**A**, **E**, **I**, **M**, **Q**), C1q (**B**), C5b-9 (**F**), CD59 (**J**), C3a (**N**) and C5a receptor (**R**) proteins were investigated by double-label immunofluorescence and confocal microscopy as detailed in Materials and Methods. TO-PRO-3 was employed to counterstain nuclei (blue; **C**, **G**, **K**, **O**, **S**). Merged images (yellow; **D**, **H**, **L**, **P**, **T**) demonstrated the co-localization (green) of C1q (**D**), CD59 (**L**), C3a (**P**) and C5aR1 (**T**) with PTX3, whereas no co-localization was evident for C5b-9 (**H**). Bar length = 50 µ.

Finally, the deposits of C1q along with CD59, C3aR and C5aR1 expressions in the initial apparently negative biopsy were confirmed in the second biopsy of group A and B patients (Fig. 6A–T), suggesting that the changes observed in PBx preceded the appearance of prostate cancer and remained after the onset of the neoplastic disease.

## Discussion

The results of the present study suggest that PTX3 expression within an inflamed prostate is quantitatively, temporarily and spatially correlated with C1q expression, suggesting the activation of classical pathway of the complement system without resulting in C5b-9 activation; finally, PTX3-associated C1q deposits along with CD59, C3aR and C5aR1 increased expression, are strictly correlated with the subsequent development of prostate cancer.

Prostate cancer remains one of the most common cause of cancer-related death in men^32^. A timely diagnosis is therefore a paramount need in its management. PSA represents the only non-invasive tool in our hands for prostate cancer screening, although the limits of this biomarker are well known, in particular the lack of accuracy and the high false positive rate^33^. We have already demonstrated the potential role of PTX3 as a tissue and serum biomarker that can reliably predict the development of prostate cancer^7^. There might be two potential limits in the development of PTX3 as a prostate cancer specific biomarker: its potential expression in other neoplasia and its association with an inflammatory status. The latter might be easily overcome by contextually measuring CRP serum levels, as we did in the present study. The former might be more relevant, although the role of PTX3 in the onset and progression of neoplasia is still controversial^34^.

Our previous results, confirmed by other studies, underlined the role of PTX3 in carcinogenesis, suggesting that this long-pentraxin may represent a key link between inflammation and neoplasia^3,7,9,35,36^. In this context, the close relationship between the Complement system and PTX3 may represent an interesting pathogenic mechanism, although experimental data on the activation of the Complement cascade in prostate cancer are lacking.

PTX3 has been shown to bind C1q as well as MBL inducing the activation of the Complement cascade^37^. In our setting, we demonstrated a clear co-localization with C1q, but not with MBL. These evidences could suggest the local activation of the Complement system through the classical pathway. Interestingly in our setting, we did not observe the formation of the terminal Complement complex that might lead to the subsequent lysis of the neoplastic cells.

C1q deposition in cancer has been associated with angiogenesis other than to complement activation^10^. Moreover, PTX3, by recruiting Factor H or inhibiting angiogenesis, can also lead to a reduced inflammatory response, carcinogenesis and angiogenesis in other tumor contexts^37,38^ and in this scenario the binding to C1q could reduce complement activation^5^.

In our data, we observed that the increased expression of C1q was associated to an increased deposition of the terminal Complement complex C5b-9 in those patients who were diagnosed with prostate cancer at the second biopsy. As in most neoplasia, transformed cells may activate several mechanisms to escape Complement-dependent lysis. In particular, the production of CD59 or protectin, one of the main inhibitors of C5b-9 assembly. This protein ubiquitously expressed at low levels in normal conditions is significantly increased in numerous neoplasia^39^. Indeed, we observed a clear up-regulation of CD59 in the patients who will subsequently develop prostate cancer both at the first and at the second biopsy. This is one of the main novelties of our findings compared to the existing literature. It is in fact well documented that cancer cells tend to upregulate various complement receptors and membrane bound complement inhibitors, including CD59 but also CD46 and CD55^10,31^; interestingly, our study is the first, to our knowledge, demonstrating an up-regulation of CD59 in a tissue before the onset of the neoplastic disease. Thus, our observation supports the hypothesis that the activation of the coagulation cascade and the inhibition of the terminal Complement complex C5b-9 may play a key role in the escape from immunosurveillance of neoplastic cells and might represent the first step in the development of clinically evident neoplastic disease. The factors modulating CD59 expression in this setting remain to be clarified, although the inflammatory milieu may represent the answer also for this event, since Bjorge et al. reported that two key pro-inflammatory cytokines, interleukin-1 and tumor necrosis factor alpha, induce the expression of CD59 in human colonic adenocarcinoma cells^39^; it is also to clarify if the classical pathway (C1q)-mediated complement activation in these patients is also characterized by other factors that can inhibit C3, C4 and C5 deposition. However, our results point out that in patients who will subsequently develop a prostate cancer the prostate tissue is characterized by the increased expression of C1q and reduced activation of the Complement system, terminally inhibited by CD59.

However, the priming of the Complement cascade, even if incomplete, is still able to release a number of mediators that may potentially have a significant role in the pathogenesis of cancer. In particular, the enzymatic digestion of C3 and C5 produces, during the activation of the Complement system, two active soluble components, C3a and C5a, respectively^40^. These two molecules have been shown to induce an array of pro-neoplastic effects. Indeed, both anaphylatoxins have been shown to promote neoplastic cell survival, proliferation and motility though the interaction with two specific cell-surface receptors, C3aR and C5aR1^31^. On the other hand, C5a can activate myeloid-derived suppressor cells inducing the production of highly immunosuppressive reactive oxygen species (ROS) and reactive nitrogen species (RNS) that, in turn, might inhibit the anti-tumor T-cell response^41^. In addition, Nabizadeh et al. recently reported that C3aR contributes to melanoma carcinogenesis through the inhibition of neutrophils and CD4^+^T cell response; they observed that in mice lacking C3a receptor the development of melanoma is significantly delayed and this effect was associated with a significant increase in infiltrating neutrophils and CD4^+^T cells^42^. moreover, Complement anaphylatoxins can trans-differentiate several resident cells^43–45^. In our setting, both C3a and C5a receptors were dramatically up-regulated in the prostate tissue of patients who will develop a prostate cancer, supporting the hypothesis that the two soluble modulators available in situ after the activation of the Complement cascade may play a direct effect on resident cells to promote carcinogenesis.

The main limit of our study, in consideration of its clinical nature, is represented by the fact that we can only demonstrate an association between PTX3 expressions and Complement activation, not a cause-effect relationship. However, our study design represents a key strength, since it allows us to provide completely novel information on the timing of Complement system activation and inhibition in the development of neoplasia.

## Conclusions

Our data support the hypothesis that in prostate cancer PTX3 may play a role in the priming of the Complement enzymatic cascade. Our findings shed light on a potential molecular mechanism linking prostate inflammation and carcinogenesis and provide solid grounds for the definition of predictive markers and the identification of specific therapeutic targets to prevent the development of prostate cancer.

## Data Availability

The datasets used and/or analyzed during the current study are available from the corresponding author on reasonable request.

## Abbreviations

**ASAP**: Atypical small acinar proliferation
**PBx**: Prostate biopsy
**PCa**: Prostatic cancer
**PIN**: Prostatic intraepithelial neoplasia
**BPH**: Benign prostatic hypertrophy
**PTX3**: Pentraxin-3
